# Evaluation of suicide among Iranian ex-prisoners of war in Iraq detention camps (1980-90)

**DOI:** 10.5249/jivr.v15i1.1783

**Published:** 2023-01

**Authors:** Saadat Soheil, Ali Khaji

**Affiliations:** ^*a*^ Sina Trauma and Surgery Research Center, Sina Hospital, Tehran University of Medical Sciences, Tehran, Iran.; ^*b*^ Iranian Research Center on Aging, University of Social Welfare and Rehabilitation Sciences, Tehran, Iran.

**Keywords:** Iraq-Iran War, Iranian Prisoners of War (POWs), Suicide

## Abstract

**Background:**

Prisoners of war (POWs) are usually at risk of suicide due to problems such as torture, social and emotional deprivation, etc. The present study aimed to investigate suicide cases among Iranian prisoners of war (POWs) over ten years of their presence in the camps in Iraq (1980-1990).

**Methods:**

Data required in this study were collected in two ways: 1- Iranian ex-POWs' death certificate by the Iraqi army clinic setting; 2- we interviewed 19 Iranian ex-POWs with sufficient information from detention camps and their events. The collected data were age, sex, duration of captivity, date of death, the suicide, and places of suicide (camp name).

**Results:**

During eight years of the Iraq-Iran war, about 40000 Iranian soldiers captured by Iraqi sol-diers. Of them, at least 11 persons (0.0275%) lost their lives due to suicide. The rate of suicide among Iranian ex-POWs in Iraq was variable from zero to 28 per 100000 people. Nine (82%) of deceased were among registered prisoners of war, and three (%27.3) were civilians. The highest rate was among prisoners that spent seven years of captivity. The most common method of suicide was hanging and burning, with 45.5% (5/11) and 18.2% (2/11), respectively.

**Conclusions:**

Social support and providing suitable treatment for chronic and incurable diseases or creating situations for returning such patients to their home could be essential for suicide prevention. Transferring them to a third country (under the supervision of international groups such as International Committee of the Red Cross (ICRC) could be another way to reduce the amount of psychological stress and will also be helpful in their treatment.

## Introduction

Suicide is a deliberate act aimed at harming yourself to death. A suicide attempt is when a person intentionally harms herself/himself but does not lead to her/his death.^1^ Suicide death is an important health problem in the world. It is estimated that about 800000 people died due to suicide each year. For every death, due to suicide, it is possible that there are 20 attempted suicides.^2^ That is why some countries have developed national suicide prevention strategies.^3,4^ During the war and military conflicts, death from suicide shows an increasing trend,^5,6^ but in military situations, especially in recent decades, more attention has been paid to deaths from suicide among on-duty personnel and soldiers that return home from war.^4,7,8^ Apart from these groups and among war victims, it seems that prisoners of war are more at risk than others.^9^ For prisoners of war, the situation is similar to soldiers.^10^ Related reports investigate suicide rates and manners after their freedom and when they returned home; however, investigations of suicide deaths in detention camps and captivity are limited.^11^ Since suicide is considered an unforgivable sin in Islam, a person who commits must either not have normal mental health or suffer unusual and extremely stressful conditions. Meanwhile, knowing the manner and cause of suicide is necessary for the implementation of preventive strategies in similar condition.

In 1980s, Saddam began a war against Iran that lasted eight years. After the ceasefire, exchanging prisoners of war took two years. The main duration for captivity was ten years, but it varied from two to ten years.^12^ During the war, about 40000 Iranian soldiers and civilian were captured and transferred to prisoners’ camps by Iraqi forces.^13^ Iranian Ex-POWs experienced severe conditions in Iraqi detention camps.^14,15^ They were in about 20 camps in the north, the west, and the center of Iraq. More than 50% of them were not registered by ICRC delegations until repatriation in August 1990.^12^ In this report, we aimed to investigate the suicide rate among Iranian POWs in Iraqi detention camps (1980-90).

## Methods 

During the Iraq-Iran war (1980-1988), about 40000 Iranians were captured by Iraqi soldiers and sent to detention camps. They were almost militants arrested during battles; meanwhile, nearly 3000 Iranian civilians in the border area were among them, too. Prisoners’ camps in Iraq concentrated mainly in three regions: 1-Mosul in the north (four camps); 2-Ramadi in the west (six camps); 3-Tikrit (10 camps); and one in Baqubah. The unregistered ex-prisoners of war were mainly in the camps of Tikrit and Baqubah region, but registered ex-prisoners of war were in the camps of Mosul and Ramadi.^12^ In this study, we used two sets of data to investigate the suicide pattern among Iranian prisoners of war in Iraqi detention camps. First: the death certificates of 574 Iranian POWs in Iraq prepared by the Iraqi Army health unit had been delivered to the Iranian authorities to Red Cross delegations. The results of this review have been reported in 2009.^12^

Second: we asked the people in charge of managing the detention camps to work with us on this study. These people include individuals selected by Iranian prisoners to run the camp and Iranian prisoners who were responsible for providing healthcare services to other prisoners. So, we interviewed a number of Iranian ex-POWs who had satisfactory information from detention camps and their events. These individuals were selected using the snowball sampling method. Finally, 19 ex-prisoners were interviewed. We used a questionnaire for the interview. At the time, they were asked to give their opinion about information recorded in the death certificates of Iranian prisoners of war issued by Iraqi Army health unit. Interview data were extracted manually from a database including the following data: name, age, sex, captivity duration of POW who committed suicide, date and place (camps) of suicide, and the methods and the cause of suicide. The software SPSS 21 was used for data analysis. 

## Results

**Figure 1:**
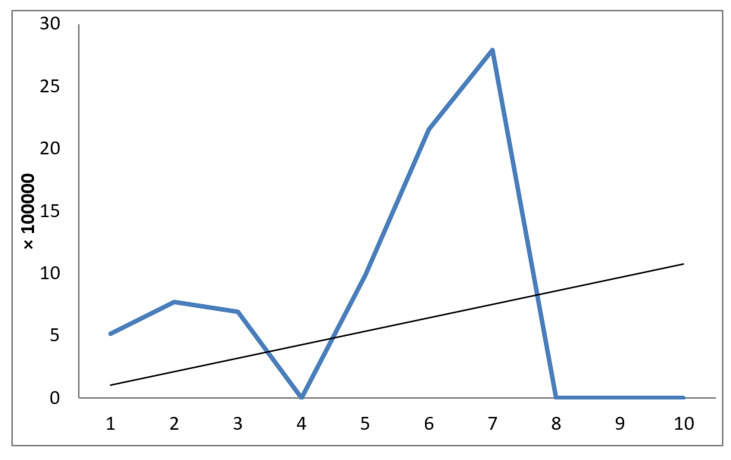
The rate of suicide death among Iranian prisoners of war in Iraqi detention camps (1980-90).

**Figure 2:**
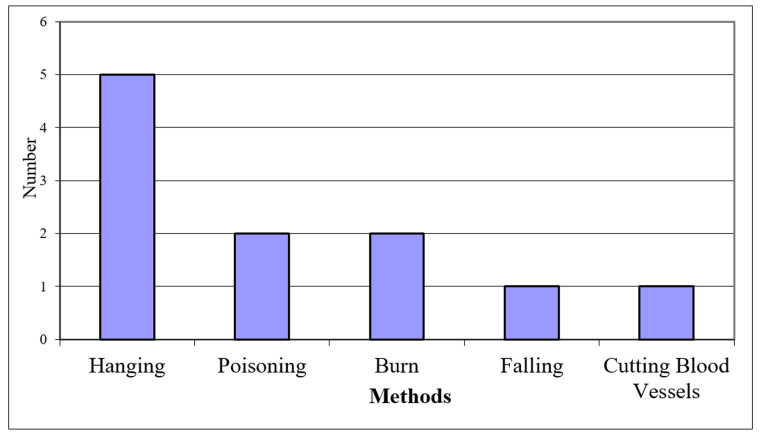
Frequency of suicide among Iranian Ex-POWs in Iraqi detention camps due to suicide methods (1980-90).

Of 40000 Iranian prisoners of war who were at least two and had ultimately ten years in Iraqi detention camps, 11(0.028%) individuals lost their lives resulting from suicide (1980-90). This comprises 0.028% of our POWs in Iraq (1980-90). The mean age of cases at the time of captivity was 21.8+2.18 (range: 20-27; Min=20). More than half of the deaths happened in Ramadi Region camps. Nine (82%) of the deceased were among registered prisoners of war. Characteristics of cases are shown in Table 1 . Three of the inmates who died from suicide were civilians, and nine were registered by ICRC. The mortality rate ranged from 0 to 28 per 100, 000 in years of captivity. The highest rate of 20 per 100,000 was detected in the 7th year of captivity (Figure 1). The most common manner of suicide was hanging with 5 (%45.5) cases (Figure 2). Due to the lack of access to their medical records, stating about underlying causes of suicide attempts is impossible, but our results show that from 11 of our cases, two suffered from psychological illness; one was suffering from disabling diseases, one another sustained long time torture, and finally, one has been abandoned by other prisoners. 

**Table 1:** Characteristics of suicide Iranian Ex-POWs in Iraqi detention camps (1880-90).

	Number (%)
**Age (median)**	21.8+2.18
**Registration**	
Registered	9 (81.8)
Unknown	2 (18.2)
**Camp Region**	
Mosul	3 (27.3)
Ramadi	6 (54.5)
Tikrit	2 (18.2)
**Military**	
Civilian	3 (27.3)
Army	3 (27.3)
Bassij	3 (27.3)
Unknown	2 (18.2)

## Discussion

Suicide is a complex and multi-factor phenomenon that has an increasing trend in the world. Biological and psychological factors play a significant role in this phenomenon.^2^ Due to the lack of information, in the present study, we only report the attempted suicide that resulted in the death of inmates. Our results show that 11 Iranian POWs (0.027%) died of suicide in Iranian camps in Iraq. The rate of suicide deaths among Iranian prisoners in Iraq ranged from zero to 28 per 100,000 people based on the years of captivity, the highest of which has returned to a group that has been seventy years since their captivity. It seems prolonged captivity and lack of hope for freedom were strong factors for suicidal behavior. Unfortunately, brief and limited reports on the suicide of prisoners are available.^16^ According to a report published in the Washington Post on 1 November 2005, 22 prisoners of war have attempted suicide 36 times. The researchers have not discussed the causes and consequences in this report and suicide reports in the Bagram Prison in Afghanistan.^17^ Among the reports on the prisoners of war in the Second World War, we are dealing with cases of collective suicide by Japanese soldiers (after surrendering their country to the United States and the Allies). This behavior was not only blamed on the culture and thoughts governing the Japanese army at the time but rather a courageous act as a behavior that resulted from intolerance of failure and was not related to the conditions of captivity. In other words, they believed that they preferred death to the disadvantage of accepting defeat. In Japanese culture, this practice has a long history and dates back to the era of the Samurai, referred to as "Harakiri".^18^ In the report of mortality death of Russian prisoners of war in Finland, suicide was reported as the cause of death for 20 (%0.10) of them (19085 mortality). For civilians in the Karelia camp, it is two persons among 4174 mortality (%0.05).^19^

Based on what was mentioned earlier, unfortunately, cited reports of the rate and causes of suicide among prisoners of war (during captivity) are unavailable. In such circumstances, perhaps the closest demographic group to the prisoners is soldiers on the battlefield. Comparing the suicidal mortality rate of soldiers to the general population accounts for a significant factor for researchers. The reports show that the suicide mortality rate of deployed soldiers from countries is different from that of the general population in their country.^20,24^ According to the study of Kaplan and colleagues, mortality from suicide among American Veterans was more than the general population. In addition, the risk of natural (disease) and external causes was equal among the two groups.^25^

We see a diversity in the rate of suicide-related mortality in Iran. Hassanian-Moghaddam et al. reviewed reports of suicidal deaths in the Iranian general population for 20 years. According to their report, the mortality ratio was ranged from 3.2% to 38% showing a considerable difference which is more than 11.8 times. The highest rate is 6.2 per 100,000 in 2003.^26^ Haghparast-Bidgoli et al. also reported an average rate of 5.5 per 100,000 for suicide mortality in the Iranian general population.^27^

The average age of those who died of suicide (excluding one person) is 22 years. In other words, they generally lived in their third decade, which is in line with the reports published on Iranian society, as most of the studies on suicide in Iran show that most people are in the 20-30 age group.^28^ Of course, it should be noted that about 85% of Iranian captives at the time of the captivity of equal age or less than 25 years old, and only about 5% of them were over 40 years old, so the population distribution that exists in the age groups of the general population is not seen among the captives.

It is reported that hanging was the most common way of suicide in Nazi Concentration camps (Konzentrationslager; KL). Other methods were cutting blood vessels, poisoning, contacting with electrified wire, and starvation.^29,30^ In a study by ElNour et al.,^31^ the use of firearms and hanging weapons with 90% and 83%, respectively, are the main methods of suicide attempts. Due to easy and constant access to weapons, it seems that the use of firearms is the most common method of suicide.^32,33^ Among war veterans, hanging is the most common method of suicide.^34^ Studies in Iran show that the four methods of hanging, poisoning, self-immolation, and firearms were the most common methods of suicide among Iranian veterans.^35^ Risk Factors for suicide in deceased Iranian veterans were investigated by Tavallaii and his colleagues. Of the 1463 deaths, 70 (4.9%) died of suicide. The most common suicide attempt was hanging (27.1%). Four people (6.6%) had a captivity history.^36^

The results of our study show that 45.5% (5/11) of detainees hanged themselves among Iranian prisoners. This is similar to reports for the general population in Iran.^37^ The method of suicide for two others was self-immolation. These two mentioned prisoners were civilians and citizens of southern cities of Iran. Self-immolation is the main method of suicide in the south and the west of Iran.^38^ Perhaps this similarity is an indication of the impact of cultural issues on choosing the method of suicide.

In various reports, having a history of psychological diseases such as depression and PTSD is also reported as a risk factor.^39,40^ It is not possible to investigate this issue among Iranian prisoners due to the lack of access to their medical records. However, according to the collected data, at least two of the missing prisoners had a history of psychiatric illnesses. Moreover, one of them had been abandoned by other prisoners of war since they believed that he was an informer for Iraqi soldiers. So, other captives did not have a friendly relationship with him as punishment. It is possible that the situation disrupted his psychological balance.

One of the victims had been tortured for a long time. According to our information, for one year (perhaps every day), he was beaten up, and the attempts to end the situation were unsuccessful until he committed suicide.

The presence of disabling diseases is another factor in suicidal attempts.^41^ One of the Iranian prisoners of war who committed suicide suffered from kidney disease for many years and always complained of pain.

The results of this study show that 3 (27.3%) of inmates who committed suicide had a history of suicide during captivity. A history of a suicide attempt is one of the risk factors for suicide. Various studies have shown that suicide attempt is one of the most lethal risk factors for completed suicide,^42^ so psychological evaluation is essential for them. Though in captivity no attention is paid to these issues.

As shown in Fig 1, the highest number of suicides occurred in the seventh year of the war, then declined sharply and reached zero in the eighth year. This time coincides with the adoption of Resolution 598 which led to a ceasefire and the end of the war. The hope for freedom and the ending of captivity can be considered the main causes of this decline. The condition for missing (non-registered) prisoners was worse than registered, so it was expected that the suicide rate for former be more than for the latter. Meanwhile, about 56% of the Iranian prisoners were missing,^14^ but the highest rate of suicide has taken place among Iranian registered inmates. This difference could be due to the following: 

1. The captivity of most missing persons was about 26 months if the average length of captivity for registered prisoners was about 4.5 years. 

2. Most of the missing persons were captured by the enemy during the final months of the war, at the time of the adoption of Resolution 598 (July 1988), so when they arrived at the camps, the war had been over. In other words, their hope for freedom and returning to their homeland was high.

3- Our knowledge about Iranian missing ex-POWs is limited, so it is possible the suicide rate for them is underestimated. 

**Limitations**

In this study, we faced two limitations: 

First: It is possible that the number of suicide presented in this study are lower than the actual one, because about thirty years have passed since then, and it is not possible to access all the necessary information in this regard. 

Second: Our information about the health condition of deceased is limited due to a lack of access to their health records. This limits our judgment of the characteristics of this behavior among Iranian POWs in Iraqi detention camps.

## Conclusion

It is important to note that the conditions in the detention camps are quite different from ordinary prisons where the time of duration of imprisonment and Prisoners' rights are well defined so, the cause and methods of suicide could be different and also prevent it. It is recommended following measures for the reduction of suicide and its deaths for prisoners of war in detention camps:

1- Further monitoring of countries' treatment of prisoners of war and their performance in concentration camps to reduce physical and psychological stress is one of the measures that can reduce the number of suicides and consequent deaths.

2- Exchanging prisoners suffering from chronic and incurable diseases can be one of the measures. If it is not possible to exchange these people, transferring them to a third country (under the supervision of international groups such as ICRC (International Committee of Red Cross) will reduce the amount of psychological stress by increasing their life expectancy and freedom and will also help in their treatment.^43^
